# Painting a specific chromosome with CRISPR/Cas9 for live-cell imaging

**DOI:** 10.1038/cr.2017.9

**Published:** 2017-01-13

**Authors:** Yuexin Zhou, Ping Wang, Feng Tian, Ge Gao, Lei Huang, Wensheng Wei, X Sunney Xie

**Affiliations:** 1 Biodynamics Optical Imaging Center (BIOPIC), School of Life Sciences, Peking University, Beijing 100871, China; 2 Beijing Advanced Innovation Center for Genomics (ICG), Peking University, Beijing 100871, China; 3 State Key Laboratory of Protein and Plant Gene Research, School of Life Sciences, Peking University, Beijing 100871, China; 4 Peking-Tsinghua Center for Life Sciences (CLS), Academy for Advanced Interdisciplinary Studies, Peking University, Beijing 100871, China; 5 Center for Bioinformatics, School of Life Sciences, Peking University, Beijing 100871, China; 6 Department of Chemistry and Chemical Biology, Harvard University, Cambridge, MA 01238, USA

## Dear Editor

Visualization of chromosome shapes and dynamics in a live cell is highly desirable and necessary in many areas of cell biology. For example, the copy number of a particular chromosome in cancer cells is often abnormal (e.g., more than two), and therefore probing chromosome copy numbers can aid cancer diagnosis. During interphase, each chromosome exists in its own territory in the nucleus, which can be imaged by fluorescence *in situ* hybridization (FISH) using sequence-specific probes of different colors^1,2^. However, such chromosome painting has only been possible in fixed cells, and is not suitable for dynamic monitoring of live cells. Therefore, it would be valuable to visualize DNA replication of one chromosome during interphase, and follow chromosome dynamics in the M phase.

Recent development of clustered regularly interspaced short palindromic repeats/CRISPR-associated proteins (CRISPR/Cas)^3^ has provided a powerful tool for live-cell imaging of genomic loci^4^. In particular, the nuclease defective Cas9 (dCas9) fused with enhanced green fluorescent protein (EGFP) is used to target a particular DNA sequence upstream of a protospacer adjacent motif (PAM) sequence. Such targeting is achieved through Watson-Crick base pairing of ∼20-bp single-guide RNA (sgRNA) that is pre-complexed with the dCas9-EGFP protein. The targeted loci can thus be fluorescently labeled in live mammalian cells^5,6,7,8^. However, the labeling achieved by this method is usually restricted to the genomic loci that consist of repetitive sequences, and has not been attempted to track an entire chromosome in a live cell.

**Figure 1:**
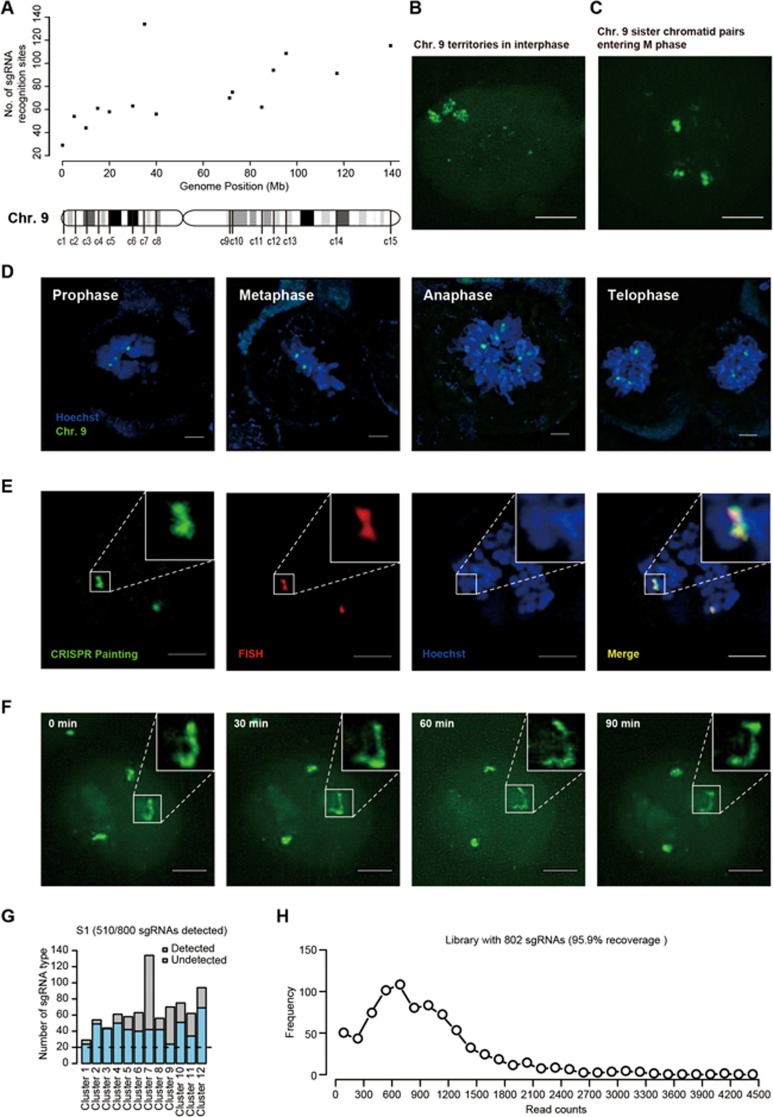
Construction of the CRISPR/Cas9 imaging system for fluorescent labeling of a particular chromosome in live cells. **(A)** Scatter plot for numbers of sgRNA-binding sites in each cluster of 5 Kb width across human chromosome 9. In the model of sgRNA-binding sites on the entire chromosome 9, vertical lines labeled as c1-c15 indicate the locations of 15 sgRNA clusters' binding sites. **(B)** Maximum intensity projection of EGFP images recorded by N-SIM at the S phase of the cell cycle. Step size, 0.12 μm; Scale bar, 5 μm. Three copies of chromosome 9 in a HeLa cell are clearly visible. **(C)** Maximum intensity projection of EGFP images recorded by N-SIM in the prophase of mitosis. Step size, 0.12 μm; Scale bar, 5 μm. Two sister chromatids of each chromosome 9 are clearly resolved. **(D)** EGFP image (green) in live cells at different stages of the M phase, recorded by an Olympus inverted wide-field fluorescence microscope. Three pairs of the chromosome 9 in the prophase and metaphase, whose two sister chromatids are not clearly resolved due to the reduced resolution, separate in the anaphase and telophase. Cells are also stained with Hoechst (blue) for DNA. All step size, 0.2 μm; depth of maximum intensity projection for prophase, metaphase, anaphase and telophase, 4 μm, 7 μm, 8 μm and 6 μm, respectively; scale bar, 5 μm. **(E)** Co-localization of chromosome 9 labeled by dCas9-EGFP (green) and C9-1 and C9-2 loci labeled by Cy3-tagged FISH probes (red). Scale bar, 5 μm. **(F)** Dynamics of the three copies of chromosome 9 in S phase in a live HeLa cell. Z maximum intensity projection of 10.5 μm. Scale bar, 5 μm. See also Supplementary information, Movie S1. **(G)** Distribution of sgRNA sequences from a single clonal sample detected by DNA sequencing after PCR amplification using the common primers for the sgRNA sequences. Single clone 1 (S1) was selected from a cell pool that has been painted with 802 sgRNAs. Majority of the sgRNA sequences have been incorporated into a cell single clone. The horizontal dashed line indicates the level with 20 sgRNA sequences per cluster, which is necessary for effective chromosome painting. **(H)** Distribution of numbers of reads for different sgRNA sequences, showing efficient incorporation and even distribution of the pooled 802 sgRNA library in the cell population.

Here we report the specific labeling of a large number of loci in the genome, which makes it possible to paint an entire chromosome in a live cell. To do so, we designed a new strategy using a large number of sgRNAs targeting mainly the non-repetitive regions of the chromosome (Figure 1A and Supplementary information, Figure S1A). To design sgRNAs, we scanned the sequence of the entire chromosome 9 on human reference genome hg19. Each 19-23 bp genome sequence upstream of a PAM sequence NGG was taken as a candidate target region. Because the efficiency of sgRNA binding is dependent on its GC content^9,10^, sgRNAs with GC content of 45%-65% were selected (Supplementary information, Figure S1B). The sgRNAs that could also bind to other chromosomes were removed in order to assure labeling specificity and reduce fluorescent background. Additionally, when multiple targeting sequences overlapped in a chromosome region, only one of them was selected. Among all sgRNAs, only one protein-coding gene (*DNAJB5*) contains sgRNA-binding sites in the exon region (Supplementary information, Figure S1C), but its expression level was not affected by lentivirus infection or dCas9-EGFP/sgRNA targeting (Supplementary information, Figure S1D).

To obtain high densities of sgRNA that would give signals significantly above the intracellular fluorescent background, especially when the chromatin is in open state during G and S phases, we chose 15 clusters of target sites (c1-c15) on chromosome 9, each spanning 5 Kb and containing more than 30 targets. The clusters are placed at least 5 Mbp away from each other (Figure 1A). In total, we selected 1 124 sgRNAs that are distributed on chromosome 9 (Supplementary information, Table S1).

As shown in Supplementary information, Figure S1A, a cell line stably expressing dCas9-EGFP was constructed from one HeLa cell by lentiviral infection. In the meantime, the 1 124 sgRNAs were packaged into lentiviruses and used to infect HeLa cells expressing dCas9-EGFP for three times. After labeling the chromosome with the 1 124 sgRNAs, we imaged cells in the S phase using a Nikon structured illumination microscope (N-SIM) equipped with a 100× TIRF oil immersion objective (NA 1.49). Figure 1B shows that the projected z-stack EGFP images. We attribute the three bright EGFP signal regions in the nucleus to three copies of chromosome 9, which is consistent with the karyotype of the HeLa cells we used (Supplementary information, Figure S1E). We noted that the EGFP signal is stronger in the S phase, when DNA replication takes place, than in the G1 phase, indicating that dCas9-EGFP can bind rapidly to newly synthesized DNA. Taken together, the imaging results suggest the efficacy of our method in visualizing a desired chromosomal territory in a live cell.

Figure 1C shows a projected z-stack of N-SIM fluorescence images taken in the prophase during mitosis. The high resolution of structured illumination microscopy and the strong signal due to chromosome condensation, allowed for the visualization of the three pairs of sister chromatids (Figure 1C) after DNA replication.

We next aimed to verify the dCas9-EGFP signals with FISH using probes targeting repetitive sequences so that a single FISH probe could access many sites. We used another two sgRNA sequences, C9-1 and C9-2, which were previously described and named^7^, to replace the three non-repetitive clusters c13-c15. C9-1 binds to a region with pericentromeric repeats on chromosome 9 and C9-2 targets 115 sites within a 5 Kb region.

We delivered 800 sgRNAs from c1-c12 clusters as well as C9-1 and C9-2 (802 sgRNAs in total) into the HeLa cells expressing dCas9-EGFP by lentivirus infection (Supplementary information, Figure S2A). Before painting the entire chromosome, we confirmed that each of the 12 clusters of sgRNAs could individually label the corresponding genomic locus efficiently (Supplementary information, Figure S2B). As expected, we observed the co-localization of dCas9-EGFP and FISH labeling of C9-1 and C9-2 (Supplementary information, Figure S2C).

After labeling the chromosome with the 802 sgRNAs, we found the imaging results for the chromosome territory and three pairs of sister chromatids are very similar to those labeled with c1-c15 containing 1 124 sgRNAs (Supplementary information, Figure S2D and E). The labeled chromosome 9 in the M phase can be easily seen even with a wide-field fluorescence microscopy. Figure 1D shows the cells in the prophase, metaphase, anaphase and telophase during mitosis. The cells were also stained with a DNA-specific dye Hoechst 33342, which allows co-localization of chromosome 9 with the rest of chromosomes. The three pairs of sister chromatids in the prophase and metaphase, though not clearly resolved with this reduced resolution, split to two sets of separate chromosomes in the anaphase and telophase. As a final control experiment, Figure 1E shows co-localization of labeled chromosome 9 of the EGFP signal with the Cy3-tagged C9-1 and C9-2 FISH probes. The fact that the EGFP areas were larger than the diffraction-limited FISH areas proves again the successful labeling of chromosome 9.

Having demonstrated stable labeling of chromosome 9, we next applied our method to dynamic monitoring using a DeltaVision Imaging System (Applied Precision/GE) equipped with a 100×/1.4 NA oil immersion objective, which allows less photobleaching, longer time for data acquisition, and confocality based on deconvolution. Figure 1F and Supplementary information, Movie S1 show chromosome dynamics at a single chromosome level for a cell in the S phase. Supplementary information, Figure S2F and Movie S2 show the dynamics of chromosome 9 during a period of 2 h from the late S phase to M phase. In addition to the three chromosome 9 spots, the nucleoli exhibit unintended EGFP signal, likely due to nonspecific binding of dCas9-EGFP protein with small RNAs in the nucleoli^8^. During the data collection period, EGFP signal from the nucleoli faded away due to the disappearance of the nucleoli in the M phase, while chromosome 9 fluorescent signal became stronger due to chromosome condensation. Supplementary information, Movie S3 shows another cell in the M phase for a period of ∼3 h. After cell division, the two daughter cells maintained strong fluorescence signals from the replicated chromosomes, indicating that chromosome labeling is kept in the daughter cells. In this movie, significant conformational fluctuation of the condensed chromosomes was observed. Although hundreds of sgRNAs were delivered by lentivirus into the cells, no obvious effects on cell proliferation were observed (Supplementary information, Figure S2G).

To evaluate how many sgRNAs were actually introduced to each cell, we carried out DNA sequencing of clonally amplified cells after PCR amplification with the common primers for sgRNA sequences^11^. Figure 1G shows the distribution of the detected 510 non-repetitive sgRNA sequences in chromosome 9 in one clonally amplified population. Single clones with higher labeling efficiency (S1) had more sgRNAs sequences incorporated than single clones with weaker fluorescence (S34) (Supplementary information, Figure S2H). Figure 1H shows the distribution of read numbers for all sgRNA sequences in the population of cells, showing that majority of the sgRNAs has been sufficiently incorporated into the cell population.

To address the question of how many sgRNAs are sufficient for painting a chromosome, we reduced the number of non-repetitive sgRNAs to 485. We found no significant deterioration of the image quality when compared with the images produced with 802 sgRNAs (Supplementary information, Figure S2I), when we imaged chromosome 9 at different phases of the M phase. Based on this, we conclude that more than 20 sgRNAs in each cluster and at least 300 types of total sgRNAs are needed in one cell for effective chromosome 9 painting. As the required number of the sgRNAs should be dependent on the length of the chromosome, we anticipate that using our design strategy ∼800 sgRNAs should be enough to label the longest human chromosome 1, which is about twice as long as chromosome 9, while ∼100 sgRNAs could be enough for the shortest chromosome. Of course, for painting other chromosomes, additional optimization and validation may be required.

In summary, by introducing hundreds of specific and non-repetitive sgRNAs in a human cell, we are able to paint an entire chromosome in a live cell for fluorescent imaging. We have visualized the spatial arrangements of homologous chromosomes and sister chromatids and tracked the movement of a particular chromosome in dividing cells. Our method will facilitate studies of functional organization of chromosomes, interactions among different chromosome regions, and long-term chromosomal dynamics in live mammalian cells.

## Supplementary Information

Supplementary information, Movie S1 Dynamics of the three copies of Chr. 9 in S phase in a HeLa cell.

Supplementary information, Movie S2 Dynamics of the three copies of Chr. 9 from late S phase to M phase within 2 hr.

Supplementary information, Movie S3 Dynamics of the three copies of Chr. 9 during ∼3 hr in the M phase.

Supplementary information, Figure S1 Design and construction of CRISPR/Casmediated chromosome painting system.

Supplementary information, Figure S2 Labeling of genomic loci by 12 clusters of sgRNA and validation of Chr. 9 labeling in live cells.

Supplementary information, Table S1 Information of 1124 sgRNAs used for Chr. 9 labelling.

Supplementary information, Data S1 Materials and Methods sgRNA design
