# Supplementary material for: Painting a specific chromosome with CRISPR/Cas9 for live-cell imaging
Source: Cell Res. 2017 Jan 13;27(2):298–301. doi: 10.1038/cr.2017.9 (PMC5339855; doi:10.1038/cr.2017.9)
Supplement: Supplementary information, Figure S1 — Design and construction of CRISPR/Casmediated chromosome painting system. [file cr20179x4.pdf]

SUPPLEMENTARY FIGURES

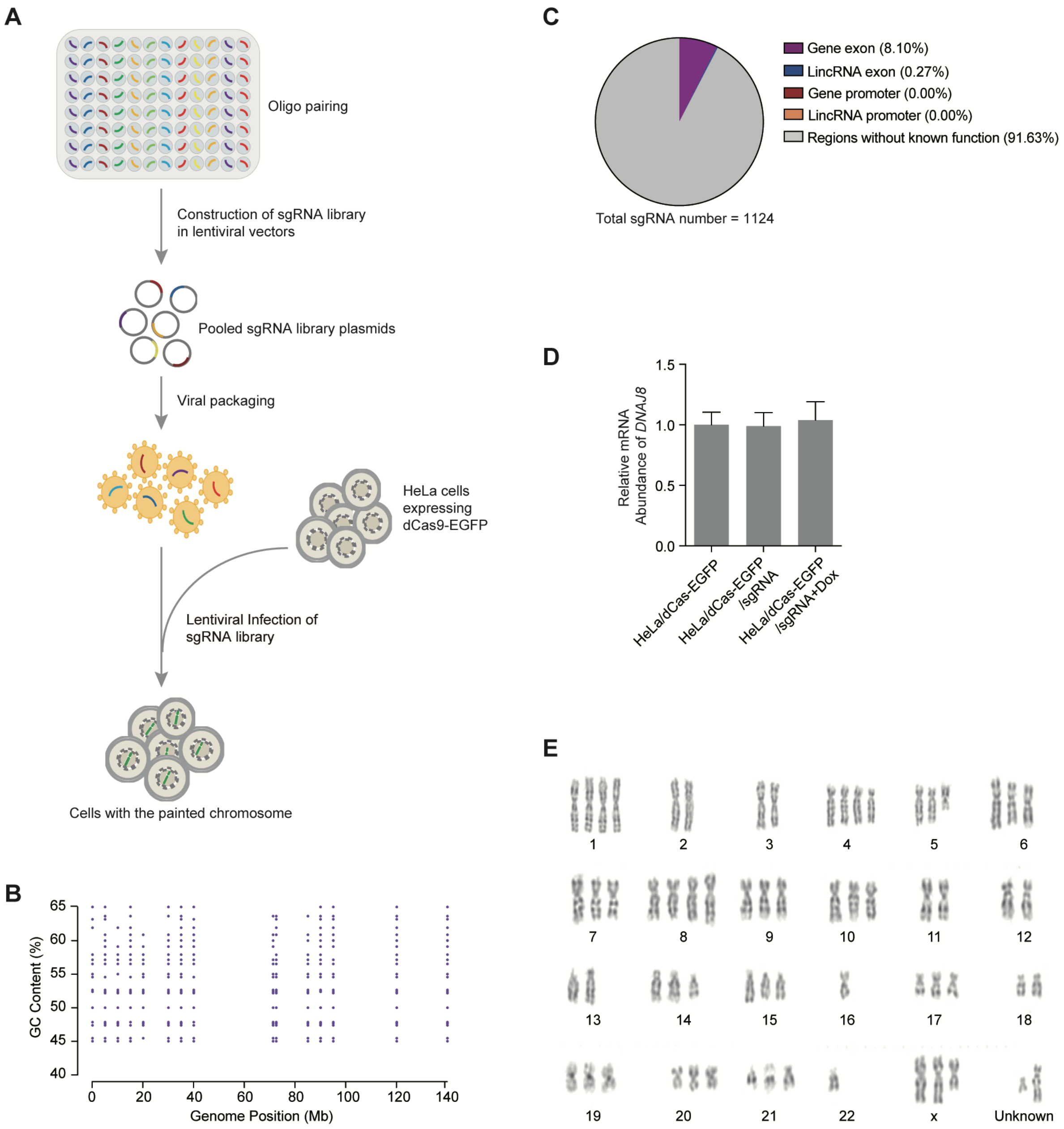

Supplementary information, Figure S1 Design and construction of CRISPR/Cas-mediated chromosome painting system. (**A**) Schematic of the CRISPR/Cas imaging system construction. Each complimentary pair of synthesized single-stranded sgRNA oligonucleotides were annealed separately. The double-stranded sgRNA oligos were ligated into the sgRNA expression plasmid. The library of plasmids of different sgRNAs were then mixed together to generate high titer lentivirus. A HeLa cell line stably expressing dCas9-EGFP was then infected three times by the sgRNA lentivirus to realize chromosome painting in live cells. (**B**) Scatter plot for the GC contents of sgRNA sequences. The x axis represents the position on human Chr. 9. (**C**) A pie chart for the distribution of sgRNA binding sites in gene context, including protein coding genes and long intergenic noncoding RNAs (lincRNAs). Most (91.63%) of the binding sites of sgRNAs were not overlapped with promoter or exon genes. (**D**) mRNA levels of *DNAJB5* (normalized to *GAPDH*) in lentivirus infected cells and Chr. 9 labeling cells, quantified by real-time PCR analysis. No obvious expression differences were observed. (**E**) Karyotype of dCas9-EGFP expressed HeLa cell line. Unknown indicates that the chromosome or segment doesn't have a typical G-bands, which is not caused by translocation of Chr. 9 with any other chromosomes.
