# Supplementary material for: Painting a specific chromosome with CRISPR/Cas9 for live-cell imaging
Source: Cell Res. 2017 Jan 13;27(2):298–301. doi: 10.1038/cr.2017.9 (PMC5339855; doi:10.1038/cr.2017.9)
Supplement: Supplementary information, Figure S2 — Labeling of genomic loci by 12 clusters of sgRNA and validation of Chr. 9 labeling in live cells. [file cr20179x5.pdf]

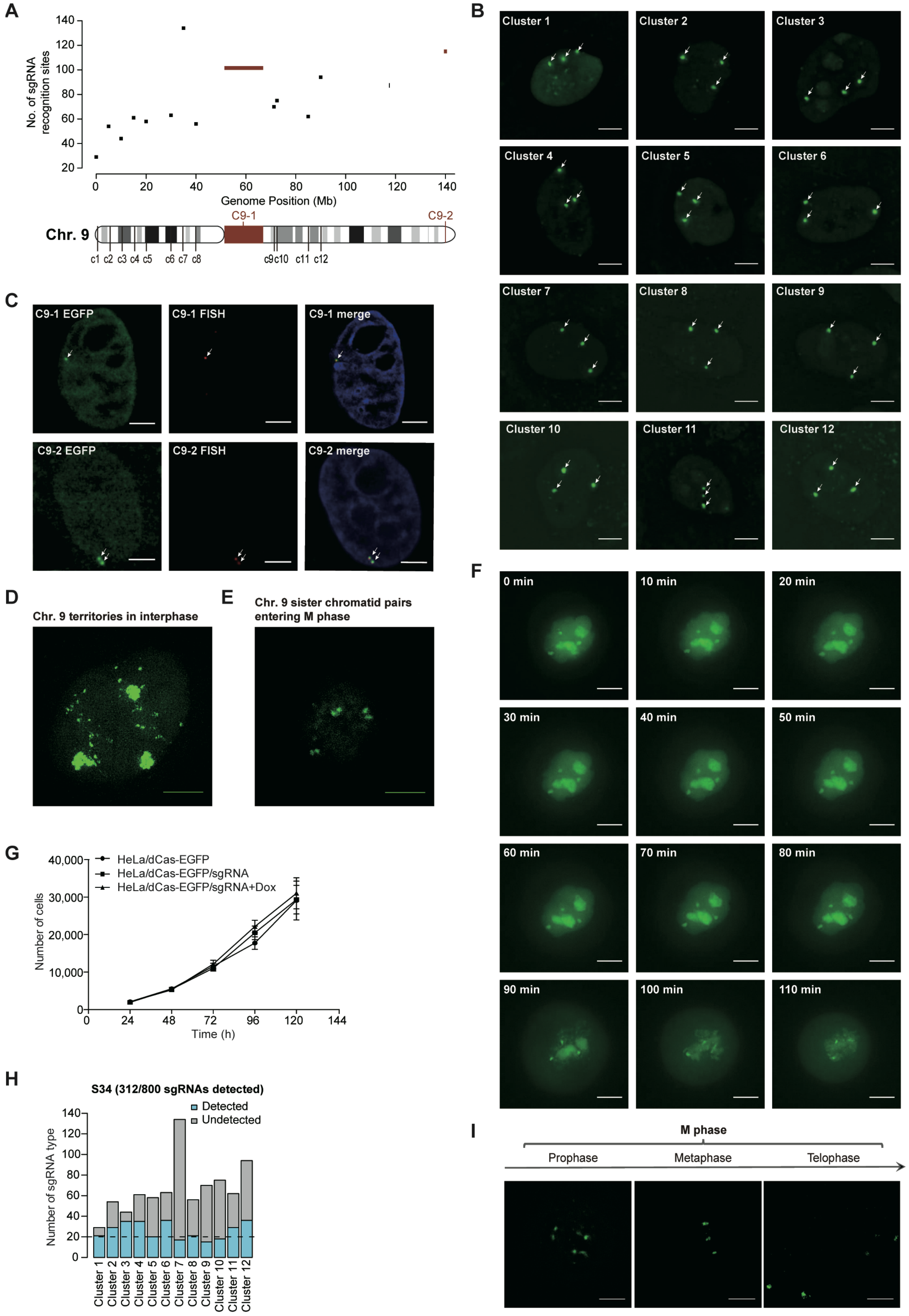
A
No. of sgRNA recognition sites
Genome Position (Mb)
C9-1
C9-2
Chr. 9
c1 c2 c3 c4 c5 c6 c7 c8 c9 c10 c11 c12
B
Cluster 1
Cluster 2
Cluster 3
Cluster 4
Cluster 5
Cluster 6
Cluster 7
Cluster 8
Cluster 9
Cluster 10
Cluster 11
Cluster 12
C
C9-1 EGFP
C9-1 FISH
C9-1 merge
C9-2 EGFP
C9-2 FISH
C9-2 merge
D
Chr. 9 territories in interphase
E
Chr. 9 sister chromatid pairs entering M phase
F
0 min
10 min
20 min
30 min
40 min
50 min
60 min
70 min
80 min
90 min
100 min
110 min
G
HeLa/dCas-EGFP
HeLa/dCas-EGFP/sgRNA
HeLa/dCas-EGFP/sgRNA+Dox
Number of cells
Time (h)
H
S34 (312/800 sgRNAs detected)
Detected
Undetected
Number of sgRNA type
Cluster 1
Cluster 2
Cluster 3
Cluster 4
Cluster 5
Cluster 6
Cluster 7
Cluster 8
Cluster 9
Cluster 10
Cluster 11
Cluster 12
I
M phase
Prophase
Metaphase
Telophase

Supplementary information, Figure S2 Labeling of genomic loci by 12 clusters of sgRNA and validation of Chr. 9 labeling in live cells. (**A**) Scatter plot for the number of sgRNA binding sites in each cluster of 5 kb width across the human Chr. 9. In the model of sgRNA binding sites on the entire Chr. 9, short horizontal lines labeled as c1-c12 indicated the locations of 12 sgRNA clusters binding locations. The red block indicated the binding sites of C9-1 and C9-2 sequences. The number of C9-1 binding sites is an approximate value. (**B**) Maximum intensity projection images of individual sgRNA clusters labeled genomic loci in live HeLa cells. dCas9-EGFP stable expressing HeLa cells were infected by individual sgRNA cluster through lentivirus infection. Images were taken by an Olympus wide field fluorescence microscope with a 0.3 μm step size. Scale bar, 5 μm. White arrows indicate the three cluster regions labeled by the sgRNAs in Chr.9. (**C**) Co-localization of dCas9-EGFP labeling (green) and FISH labeling (red) for C9-1 and C9-2 loci. Cells were stained with Hoechst 33342 (blue). Scale bars, 5 μm. (**D**) Maximum intensity projections for EGFP images recorded by a Nikon structured illumination microscope (N-SIM) at the S phase of interphase. Step size 0.12 μm, 8 μm depth. Scale bar, 5 μm. (**E**) Maximum intensity projections for EGFP images recorded by N-SIM in the prophase of M phase. Step size 0.24 μm, 11 μm depth. Scale bar, 5 μm. (**F**) Dynamics of the three copies of Chr. 9 from late S phase to prophase within 2 hr. During this time, the unintended strong EGFP signal within nucleoli decreased when nucleoli disappeared in M phase. Images were taken at 24 evenly separated time points, 12 of them were shown here, showing z maximum projection of 10.5 μm. Scale bar, 5 μm. See also **Supplementary information, Movie S2**. (**G**) Cell proliferation curve of dCas9-EGFP expressed HeLa cells, HeLa cells infected by lentivirus containing 802 different sgRNAs and Chr. 9 labeled HeLa cells. Values represent the mean ± S.E.M. of 9 independent experiments. No obvious effects on cell proliferation were observed. (**H**) Distribution of detected sgRNA sequences by DNA sequencing after PCR amplification with the common primers for the sgRNA sequences for a single clonal sample. Single clone S34 was selected from cell pool with 802 sgRNAs. The horizontal dashed lines indicate the level with 20 sgRNA sequences per cluster, which is necessary for effective chromosome painting. (**I**) Chr. 9 labeling with decreased number of sgRNAs at different stages of M phase imaged by N-SIM. Snapshots of Chr. 9 in X-Y plane were shown. The images were maximum intensity projection of 11 μm depth with 0.24 μm step size. Scale bars, 5 μm.
