# Supplementary material for: Painting a specific chromosome with CRISPR/Cas9 for live-cell imaging
Source: Cell Res. 2017 Jan 13;27(2):298–301. doi: 10.1038/cr.2017.9 (PMC5339855; doi:10.1038/cr.2017.9)
Supplement: Supplementary information, Data S1 Materials and Methods — sgRNA design [file cr20179x7.pdf]

Supplementary information, Data S1 Materials and Methods

sgRNA design

Chromosome 9 sequence information were downloaded from UCSC genome browser. Sequences in the forms of $N_{19\text{-}23}$NGG and with the GC content ranging from 45% to 65% in non-PAM regions were selected as candidate sgRNA targeting sites. sgRNA sequences were then mapped to human genome using bowtie (v2.2.5) to discard the sgRNAs which could also target to the other chromosomes. Overlapped sgRNA sequences were removed so that each binding site was covered by only one sgRNA. We placed 15 clusters of target sites along chromosome 9 (Figure 1A), each cluster spans 5 kb and contains more than 30 targets and the clusters are placed at least 5 Mbp away. Because dCas9 protein binding may affect the expression of endogenous genes, we evaluate the distribution of sgRNA binding sites in the different gene context. No sgRNAs were mapped to the -50 ~ +300 bp of the gene TSS regions, which were reported to significantly affect the expression of target genes [9]. Only one protein coding gene (*DNAJB5*) and one long intergenic noncoding RNA (lincRNA) (RP11-274B18.4) contained sgRNA binding sites in the exon region (Supplementary information, Figure S1C). The expression level of DNAJB5 would not be affected by lentivirus infection or dCas9-EGFP/sgRNA targeting (Supplementary information, Figure S1D).

Cell culture and plasmid construction

HeLa cells and HEK293 cells were cultured in Dulbecco’s modified Eagle medium (DMEM) (Gibco) with 10% FBS (Gibco) and 1% P/S (Life Technologies). All cells were cultured at 37°C and 5% CO_2 in a humidified incubator.

The lentiviral sgRNA vector was constructed by substituting EGFP gene in pLenti-sgRNA-Lib [11] for Puromycin resistant gene. The oligonucleotides for each sgRNA-coding sequence were individually synthesized (GENERAY, Inc.). Paired oligonucleotides were mixed in 96-well plates to the final concentration of 9 μM in 50 μl of 1×TransTaq HiFi Buffer II (Transgen) for annealing. Annealed oligonucleotide pairs belonging to the same labeling cluster were then mixed, phosphorylated and ligated into the lentiviral sgRNA vector using Golden Gate method. Different clusters of ligation mixtures were transformed separately into Trans1-T1 competent cells (Transgen) to obtain the sgRNA plasmids for each of the labeling clusters.

Lentivirus package and stable expression of dCas9-EGFP and sgRNAs

4×10^6 of HEK293 cells were seeded at 10 cm plate 24 hr before transfection. 0.4 μg pVSVG plasmid, 4 μg pR8.74 and 4 μg dCas9-EGFP expressed plasmid or Tet on 3G or mixed plasmids of sgRNAs were co-transfected into HEK293 cells by X-tremeGENE HP (Roche). Virus was harvested at 72 hr post-transfection then concentrated by Lenti-X Concentrator (Clontech) as the recommended protocol. For dCas9-EGFP expressed cell line construction, HeLa cells were infected by mixture of dCas9-EGFP and Tet on 3G virus. After induction of 200 ng/ml Doxycycline (Clontech), EGFP positive single clones were sorted by FACS. For chromosome labeling, HeLa cells were infected with sgRNA mixture virus for 3 times (For each time of infection, MOI = 60). The individual sgRNA cluster lentivirus was packaged in the same way as mentioned above.

Fluorescence in situ hybridization

Cells were fixed by 4% paraformaldehyde (Coolaber) for 15 min, permeabilize the cells with 0.5% NP-40 (Sigma) in 1× PBS (Gibco) for 10 min, wash samples with PBS for 5 min, then incubate cells with 2 ng/μl Cy3 labeled oligo-DNA FISH probe (GENERAY, Inc.) for 12 hr in a dark and humidified box at room temperature. Wash cells with 2×SSC (Invitrogen) three times and stain with Hoechst 33342 (Life Technologies).

FISH probe sequence (5’-3’):

C9-1: TTCCATTCCATTCCATTCCA

C9-2: GGGGAGCTTCCTCACAGACA

Cell cycle arrest

For imaging chromosome of cells in M phase, cells were seeded into a 35 mm glass bottom dish (In Vitro Scientific) and treated by 1×Thymidine (Sigma) for 23 hr, wash cells with PBS (Gibco) for three times. Cultured cells with fresh DMEM for 4-6 hr, then cultured cells with 1× Nocodazole (Sigma) for another 12 hr. For imaging interphase chromosome territories, cells were seeded into a 35 mm glass bottom dish one day in advance, and then treated by 1×Thymidine for 20 hours and released for 7 hr before imaging.

Quantitative real-time PCR (qRT-PCR)

RNA of different cells was extracted by RNAprep Pure Micro kit (TIANGEN), and the cDNA was synthesized separately by QuantScript RT kit (TIANGEN). SYBR Premix Ex Taq II

(TaKaRa) were used to perform Real-time PCR on LightCycler96 qPCR system (Roche). Expression levels of GAPDH were measured as normalized controls.
Specific primers for *DNAJB5* (5’-ACACCACAGACAAGACAGATCC-3’/5’-ATTGAGTGGAGTGAGTGTTGGG-3’) were used for qRT-PCR.

Cell proliferation assay

For cell proliferation assay, cells were seeded in 96-well cell culture dishes in 9 repeats at a density of 2000 cells per well. Cell numbers at the indicated time points was determined by MTT (3-(4,5-dimethylthiazol-2-yl)-2,5-diphenyltetrazolium bromide) assay.

sgRNA distribution analysis

sgRNA distributions in pool and clonal populations were identified by high-throughput deep-sequencing analysis as described before [11]. Briefly, the genomic DNA of two cell pools and different cell clones were isolated from 10^6 cells using the DNeasy Blood and Tissue kit (Qiagen). sgRNA-coding regions integrated into the chromosomes were PCR-amplified with 28 cycles of reaction using primers annealed to the flanking sequences of the sgRNAs (Primers sequences: 5’-TATCTTGTGGAAAGGACGAAACACC-3’/5’-AATACGGTTATCCACGCGGC-3’). Then the PCR products of each samples were purified and subjected to high-throughput sequencing analysis with Illumina HiSeq 2500.

Microscopy setup

Three different microscopes were used in our experiment. For the non-repetitive sequence single point labeling, z-scanning and C9-1, C9-2 FISH verification, images were acquired on an inverted wide-field fluorescence microscope (Olympus IX83) which equipped with a CCD camera (CoolSnap HQ2, Photometrics), a 100× UPlanSApo oil immersion objective lens (NA 1.40) and a LED light source (Spectra X Light Engine, Lumencor). EGFP was excited at 470/24 nm (wavelength/bandwidth), Hoechst was excited at 395/25 nm and Cy3 was excited at 550/15 nm.

Super resolution images were acquired on a Nikon Structured Illumination Microscope (N-SIM) with a SR Apo TIRF 100× oil immersion lens (NA 1.49), a Tokai Hit incubation chamber (at 37 °C) and an EMCCD (ANDOR iXon3). EGFP was excited at 488 nm. At the S phase of interphase, images were taken at step size 0.12 μm. At the prophase

of M phase, images were taken at a step size 0.12 μm for the 15 clusters and 0.24 μm for the 12 clusters.

For the long term time-lapse acquisition of cell division process, images were taken on a DeltaVision microscopy system with a UPlanSApo 100× oil immersion objective (NA 1.40), a CCD camera (CoolSnap HQ2) and a 37 °C stage incubator. EGFP was excited at 475/28 nm. 3D fluorescence images (2 μm step size) were collected every 3 min for about 3 hr to acquire 56 time points. For movies in interphase, 3D fluorescence images (1.5 μm step size) were collected every 5 min for about 2 hr.

Data analysis

All the wide-field fluorescence imaging data were analyzed by ImageJ. The N-SIM results were processed with NIS Element AR (Nikon). Images were projected in maximum intensity. All movies taken by DeltaVision microscopy system were deconvoluted and analyzed by SoftWoRx.
